# Y45G5AL.1 is a potential Kinesin-1 Interacting Protein on the Surface of Yolk Granules

**DOI:** 10.17912/micropub.biology.001827

**Published:** 2025-10-26

**Authors:** Denisa Lazureanu, Francis McNally

**Affiliations:** 1 Molecular and Cellular Biology, University of California, Davis, Davis, California, United States

## Abstract

In *C. elegans* , kinesin/ KCA-1 on the surface of yolk granules is thought to drive outward migration of the meiotic spindle by transporting membranous organelles inward on microtubules with minus ends at the cortex. Here we used BioID to identify Y45G5AL.1 as a potential KCA-1-interacting protein. Y45G5AL.1 co-localized with KCA-1 on 20% of membrane vesicles and a constitutively active kinesin-1 prematurely packed Y45G5AL.1 vesicles into a central ball in diakinesis oocytes. These results indicate that Y45G5AL.1 is on the cytoplasmic surface of endosomes that are direct cargo of kinesin 1.

**Figure 1. Y45G5AL.1 co-localizes with KCA-1 vesicles in oocytes:**
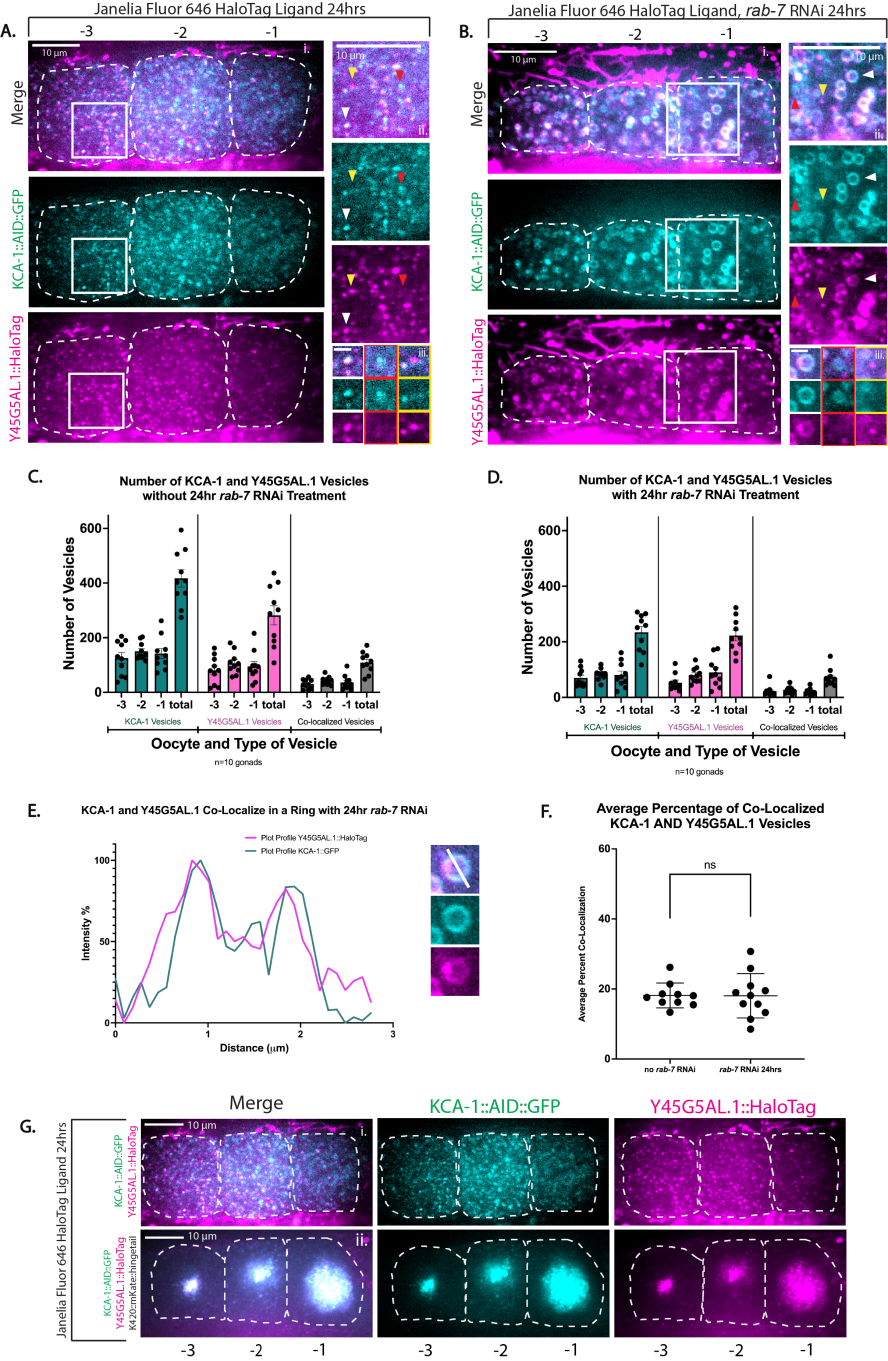
(A) Single Z-plane fluorescence microscopy images of the -3, -2, and -1 oocytes with KCA-1 ::GFP (cyan) and Y45G5AL.1 ::HaloTag (magenta) puncta. The white box in (A i.) provides a reference for the close-up image in (A ii.). Arrowheads point to different conditions of localization and co-localization that correspond to close-up images in (A iii.). The white arrow points to co-localization of a KCA-1 and Y45G5AL.1 vesicle, the red arrow points to a KCA-1 vesicle independent of Y45G5AL.1 , and the yellow arrow points to a Y45G5AL.1 vesicle independent of KCA-1 . (B) Single Z-plane fluorescence microscopy images of the -3, -2, and -1 oocytes of worms with KCA-1 ::GFP (cyan) and Y45G5AL.1 ::HaloTag (magenta) fed *rab-7* dsRNA over 24hrs. The white box in (B i.) provides a reference for the close-up image in (B ii.). Arrowheads point to different conditions of localization and co-localization that correspond to close-up images in (B iii.) The white arrow points to co-localization of a KCA-1 and Y45G5AL.1 vesicle, the red arrow points to a KCA-1 vesicle independent of Y45G5AL.1 , and the yellow arrow points to a Y45G5AL.1 vesicle independent of KCA-1 . (C) Comparision of the number of each vesicle type in the WT treatment condition for each oocyte. (D) Comparision of the number of each vesicle type in the 24hr *rab-7* RNAi treatment condition for each oocyte. (E) Overlapping plot profiles of an enlarged KCA-1 ::GFP and Y45G5AL.1 ::HaloTag vesicle showing co-localization. (F) Comparison of the co-localization percentages between the two conditions. (G) Single Z-plane fluorescence microscopy images of the -3, -2, and -1 oocytes containing KCA-1 ::GFP (cyan) and Y45G5AL.1 ::HaloTag (magenta) and compares a strain with WT kinesin-1 and a strain with the transgene expressing the K420::mKate::hingetail construct. Labeled scale bar = 10 um. Unlabeled scale bar = 1um.

## Description

Cortical positioning of female meiotic spindles is a highly conserved phenomenon that allows expulsion of extra chromosomes into polar bodies. In *C. elegans* , early spindle translocation to the cortex is dependent on kinesin 1 heavy chain ( UNC-116 ), kinesin light chains ( KLC-1 ,2) and the *Caenorhabditis* -specific kinesin cargo adapter, KCA-1 (Yang et al., 2005). UNC-116 / KCA-1 / KLC-1 is also required to pack yolk granules (McNally et al., 2010) and mitochondria (Beath et al., 2024; Aquino et al., 2025) inward and KCA-1 and KLC-1 are predominantly localized on the surface of yolk granules during outward migration of the spindle (Aquino et al., 2025). These results led to a model in which inward transport of organelles on cytoplasmic microtubules with plus ends extending inward from the cortex indirectly forces the spindle outward by volume exclusion. This model is supported by the finding that artificially coupling kinesin motor domains to mitochondria is sufficient to restore outward spindle movement in an *unc-116* germline null mutant (Aquino et al., 2025). Because KCA-1 and KLC-1 are the only proteins known to be on the surface of yolk granules, it is not known how they are targeted to yolk granules and it has not been possible to test whether artificially attaching kinesin motor domains to yolk granules is sufficient to drive outward spindle movement.

To identify potential KCA-1 interacting proteins on the surface of yolk granules, we conducted an indirect BioID experiment (Holzer et al., 2022) with KCA-1 ::AID::GFP as bait and a germline specific anti-GFP nanobody::TurboID. The most enriched prey proteins relative to a no-GFP control strain with p < 0.03 in order of enrichment were GFP, KCA-1 , SPD-5 , KLC-1 , K09G1.1 and Y45G5AL.1 . The enrichment of GFP, KCA-1 (Bait), and KLC-1 [known interactor (Yang et al., 2005)] indicated that enrichment was specific. Here we analyzed Y45G5AL.1 , a protein without homologs outside the genus *Caenorhabditis* and without predicted transmembrane or other domains. To validate the potential interaction between KCA-1 and Y45G5AL.1 , we tagged the endogenous Y45G5AL.1 locus with HaloTag. Y45G5AL.1 ::Halo and KCA-1 ::GFP both appeared in diakinesis oocytes as puncta spread diffusely across the oocyte cytoplasm ( Fig. 1A .). Depletion of RAB-7 by RNAi, which potentially blocks transport/maturation of late endosomes to lysosomes, causes vitellogenin to be trapped in enlarged vesicles (Grant and Hirsh, 1999) and KCA-1 ::GFP labels the cytoplasmic surface of these enlarged yolk granules (Aquino et al., 2025; Fig. 1B ). Y45G5AL.1 ::Halo co-localized with KCA-1 ::GFP on 20% of control vesicles ( Fig. 1A, 1C, 1F) and on 20% of enlarged *rab-7 (RNAi)* vesicles ( Fig. 1B, 1D, 1E, 1F).

To further test whether Y45G5AL.1 is a direct cargo of kinesin-1, we utilized a constitutively active form of kinesin-1, K420::mKate::hingetail, which packs yolk granules into a central ball in late diakinesis oocytes which have microtubule plus ends extending inward (Aquino et al., 2025). Whereas 10/10 control worms had dispersed KCA-1 and Y45G5AL.1 vesicles. 10/10 K420::mKate::hinge tail-expressing worms had both KCA-1 vesicles and Y45G5AL.1 vesicles packed inward into a central ball in -1 through -3 oocytes ( Fig. 1G ). Even though only 20% of Y45G5AL.1 vesicles co-localized with KCA-1 , 90% of Y45G5AL.1 vesicles were packed into a central ball by consitutively active kinesin 1. These results indicate that Y45G5AL.1 is in close proximity with KCA-1 on the cytoplasmic surface of endosomal vesicles that are direct cargo of kinesin 1.

## Methods

*Y45G5AL.1 ( syb8791 [ Y45G5AL.1 ::HALO]) V* was generated by SUNY Biotech using CRISPR/Cas9. 100 ul of 2.5 uM Janelia Fluor 646 HaloTag ligand (diluted into M9 buffer from a 200 uM stock in DMSO) was pipetted onto a 35 mm MYOB plate containing 200ug/nl ampicillin and IPTG and seeded with HT115 bacteria expressing dsRNA from the L4440 empty plasmid or *rab-7* . L4 larvae were placed on these plates for 24 hrs. For image acquisition, adult worms were anesthetized in a solution of 0.1% tricaine, 0.01% tetramisole, and 1x PBS buffer on a watch glass for 30 minutes as described in (Danlasky et al., 2020). They were then mounted onto a thin, 2% agarose pad and covered with a 22x33mm coverslip. The slides were imaged with a Yokogawa CSU-10 spinning disk confocal microscope equipped with an Olympus 100X/1.35 objective, a Hamamatsu Orca Quest qCMOS detector, a 100-mW Coherent Obis laser at 30% power, and MicroManager software. Single time point image Z-stacks were taken of the -3, -2, and -1 oocytes at 0.2 um Z intervals with a PI E662 LVPZT piezo objective collar. Exposures were 200ms for all channels and 46nm pixel size for the Orca Quest qCMOS detector. To quantify co-localization, ImageJ/FIJI software was used with the cell counter feature. All vesicles in the KCA-1 and Y45G5AL.1 channels were marked and counted separately for each oocyte. The marked vesicles were compared and analyzed for co-localization where both vesicle types overlap. GraphPad Prism was used to create graphs and for statistical analysis.

## Reagents

**Table:** 

**Strain Number**	**Genotype**	**Shorthand**	**Available From:**
**FM1178**	*kca-1 ( syb7873 [ kca-1 ::AID::GFP]) I; * *Y45G5AL.1 ( syb8791 [ Y45G5AL.1 ::HALO]) V; * wjIs76[Cn_ unc-119 (+); pie-1p::mKate2:: tba-2 ];	KCA-1 ::GFP Y45G5AL.1 ::HaloTag	fjmcnally@ucdavis.edu
**FM1223**	kca-1 ( syb7873 [ kca-1 ::AID::GFP]) I; Y45G5AL.1 ( syb8791 [ Y45G5AL.1 ::HALO]) V; ItIs44pAA173; [pie-1p-mCh::PH(PLC1delta1) + unc-119 (+)]V (no FLP) wjIs76[Cn_ unc-119 (+); pie-1p::mKate2:: tba-2 ]; pFM1944(K420::mKate::hinge tail) II;	*kca-1 ::GFP * *Y45G5AL.1 ::HaloTag * *K420::mkate::hingetail*	fjmcnally@ucdavis.edu

## References

[R1] Yang HY, Mains PE, McNally FJ (2005). Kinesin-1 mediates translocation of the meiotic spindle to the oocyte cortex through KCA-1, a novel cargo adapter.. J Cell Biol.

[R2] McNally KL, Martin JL, Ellefson M, McNally FJ (2009). Kinesin-dependent transport results in polarized migration of the nucleus in oocytes and inward movement of yolk granules in meiotic embryos.. Dev Biol.

[R3] Beath EA, Bailey C, Mahantesh Magadam M, Qiu S, McNally KL, McNally FJ (2024). Katanin, kinesin-13, and ataxin-2 inhibit premature interaction between maternal and paternal genomes in C. elegans zygotes.. Elife.

[R4] Aquino AP, Li W, Lele A, Lazureanu D, Hampton MF, Do RM, Lafrades MC, Barajas MG, Batres AA, McNally FJ (2025). Inward transport of organelles drives outward migration of the spindle during C.&nbsp;elegans meiosis.. Cell Rep.

[R5] Holzer E, Rumpf-Kienzl C, Falk S, Dammermann A (2022). A modified TurboID approach identifies tissue-specific centriolar components in C. elegans.. PLoS Genet.

[R6] Grant B, Hirsh D (1999). Receptor-mediated endocytosis in the Caenorhabditis elegans oocyte.. Mol Biol Cell.

[R7] Danlasky BM, Panzica MT, McNally KP, Vargas E, Bailey C, Li W, Gong T, Fishman ES, Jiang X, McNally FJ (2020). Evidence for anaphase pulling forces during C. elegans meiosis.. J Cell Biol.

